# Physical mechanism of spring and early summer drought over North America associated with the boreal warming

**DOI:** 10.1038/s41598-018-25932-5

**Published:** 2018-05-14

**Authors:** Woosuk Choi, Kwang-Yul Kim

**Affiliations:** School of Earth and Environmental Sciences, Seoul National University, Seoul, Korea

## Abstract

Drought during the early vegetation growing season (spring through early summer) is a severe natural hazard in the large cropland over North America. Given the recent increasing severity of climate change manifested as surface warming, there has been a growing interest in how warming affects drought and the prospect of drought. Here we show the impact of boreal warming on the spring and early summer drought over North America using Cyclostationary Empirical Orthogonal Function analysis. Northern Hemispheric warming, the leading mode of the surface air temperature variability, has led to a decrease in precipitation, evaporation and moisture transport over the central plain of North America. From a quantitative assessment of atmospheric water budget, precipitation has decreased more than evaporation and moisture transport, resulting in increased (decreased) moisture in the lower troposphere (land surface). Despite the increased moisture content, relative humidity has decreased due to the increased saturation specific humidity arising from the lower-tropospheric warming. The anomaly patterns of the soil moisture and Palmer Drought Severity Index resemble that of the anomalous relative humidity. Results of the present study suggest a credible insight that drought in the main cropland will intensify if the anthropogenic warming continues, exacerbating vulnerability of drought.

## Introduction

Surface warming due primarily to the anthropogenic forcing has been observed to be significant, particularly over the Northern Hemispheric continents. As a result of warming, global atmospheric circulations also have changed. Arctic sea ice has been melting rapidly in recent years, which serves as an additional forcing to the atmosphere^1–3^. In addition, tropical regions are believed to be expanding because of widening Hadley circulation^4–6^. The global warming has become a serious issue not only in the context of its long-term climatic effects such as addressed above but also because of its impact on the characteristics of weather extremes^7–11^. The latter affects the life on the earth significantly. Many previous studies reported the possible future increase of the risks associated with more powerful hurricanes due to increased latent heat energy from the warmer ocean, severer heat waves, and changed areas and characteristics of flooding and drought^9,11–15^. However, detailed mechanisms for changing extremes associated with warming are still uncertain^16^.

From a hydrological perspective, many global climate models predict that climatic aridity will increase in the 21st century^7,17,18^, but drought change due to warming is still an open question because it varies significantly by region and model^7,16,19^. If future drought develops over croplands such as North America during the early vegetation-growing season (spring and early summer), it can lead to a serious food problem^20–23^. Drought is not a transient episode, but rather a persistent phenomenon that is closely related to food and water security, and land surface condition if it lasts for more than a few months. Even though the central plain of North America is known as a large crop-producing region, we have little understanding of the physical mechanism of drought and how it is affected by the continuing warming. Because drought is a complicated phenomenon of air-land-hydrology interaction, its physical mechanism should be understood by comprehensive analysis of atmospheric and hydrological variables^24–27^. Moreover, drought change with warming should be indispensable, considering that warming is expected to continue in the future with a strong certainty^7,19,28^.

This study aims to understand spring to early summer drought mechanism over the central plain of North America due to the overall Northern Hemispheric warming. Changes in circulation, atmospheric moisture supply and demand will be investigated together with changes in surface moisture conditions to assess the hydrological changes due to warming. We use climate reanalysis data including 3-dimensional wind, relative and specific humidity, soil moisture, precipitation, and Palmer Drought Severity Index (PDSI) since 1979 to investigate the warming-related changes and underlying reasons. To diagnose the warming effect on drought, the cyclostationary empirical orthogonal function (CSEOF) technique and regression analysis in CSEOF space are employed. The CSEOF analysis is a powerful method of assessing the space-time evolution of climate variability and its relationship with a chosen target phenomenon^29,30^.

## Results

### Long-term changes in the NH warming-related climate variability

**Figure 1:**
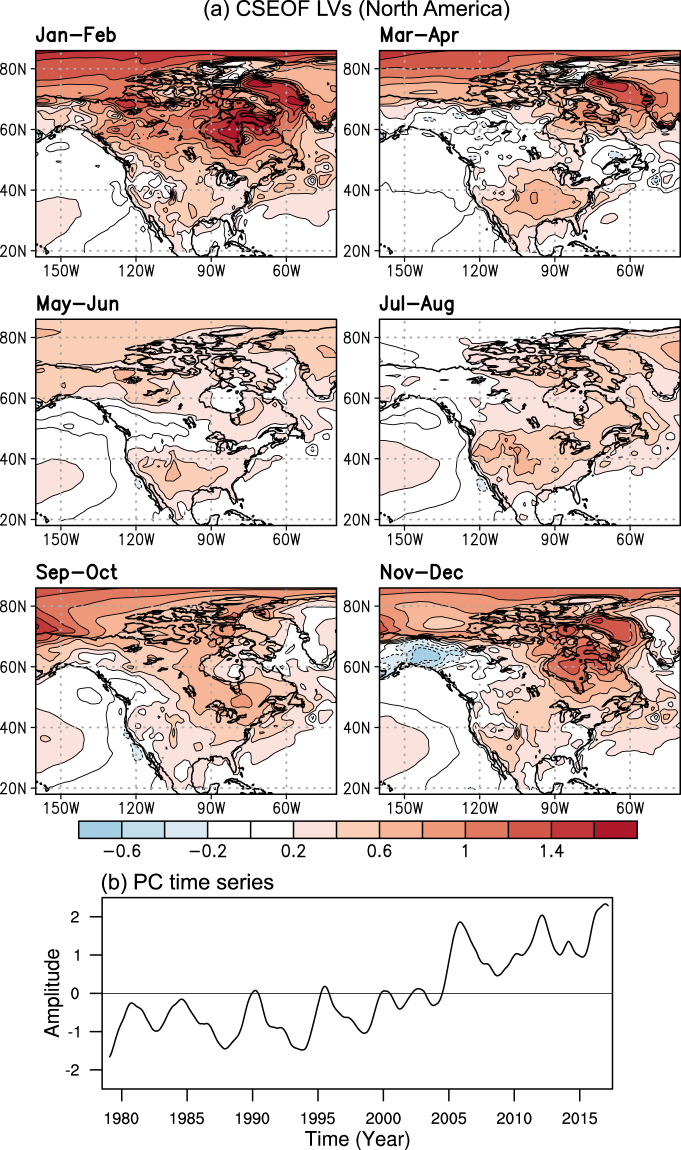
(**a**) The bimonthly averaged patterns of the loading vector and (**b**) the corresponding PC (principal component) time series of the first CSEOF (cyclostationary empirical orthogonal function) mode of 2 m air temperature over the Northern Hemisphere. This figure was created by using the Grid Analysis and Display System (GrADS) version 2.1 available at http://cola.gmu.edu/grads.

The Northern Hemispheric (NH, 18°–88.5°N) 2 m air temperature warming mode is identified as the leading CSEOF mode, and its seasonal patterns over North America are portrayed in Fig. 1. If we include the equatorial region, the NH warming mode would not be clearly identified because of the dominance of the tropical climate variability (e.g., El Niño-Southern Oscillation). Thus, the equatorial region is excluded from the analysis and we focus on the boreal extratropical region only. The first mode explains about 41% of the total 2 m air temperature variability over the NH. Combining the CSEOF loading vector and the corresponding principal component (PC) time series shows how the spatial patterns of warming have amplified in North America. The long-term warming over NH is a robust phenomenon in much of the North America region considering the overall positiveness of the CSEOF loading vector and a clear increasing trend in the PC time series. As is well known, warming in the high-latitude region (e.g., Greenland, Hudson Bay, and northern Canada) is more obvious in winter. There has been approximately 3–4 °C early growing season warming in the high-latitude region and central North America since 1979, whereas relatively weak warming less than 0.5 °C has been observed in the western Canada.

### Changes in PDSI and its association with the NH warming

The overall climatology of hydrological land surface condition has changed due to the boreal warming. It can be estimated how much the severity of droughts has changed by investigating the PDSI. The PDSI is closely related with top 1 m soil moisture, so changes in soil moisture would be one of the reasons for the PDSI pattern^31–33^. The soil moisture content, in turn, is determined by water supply and demand as well as antecedent soil conditions, water-holding capacity of the surface which is related with precipitation frequency and intensity. Even though heavy rainfall can provide lots of water in a short time, the subsurface soil might remain dry by an immediate runoff. On the other hand, long-lasting stratiform rainfall may reach subsurface soil by a weak runoff.

**Figure 2:**
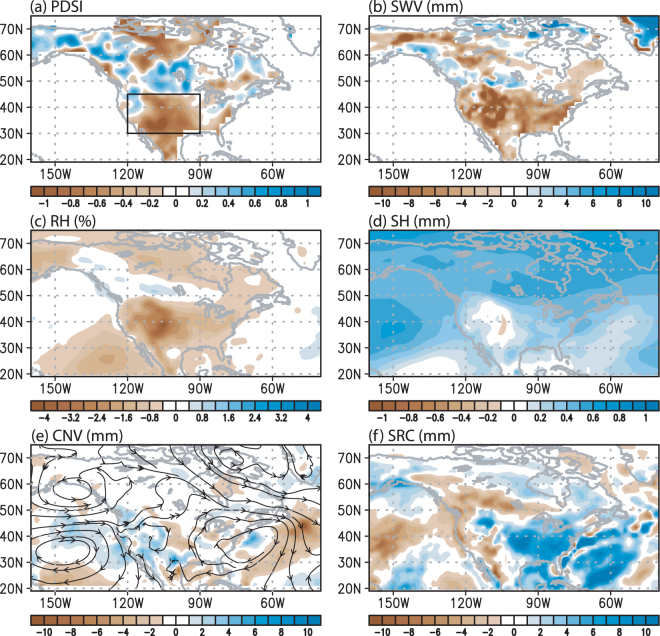
The regressed patterns of March–June averaged fields for the NH warming mode: (**a**) Palmer Drought Severity Index (fraction), (**b**) soil water volume (mm), (**c**) lower-tropospheric (1000–800 hPa) mean relative humidity (%), (**d**) lower-tropospheric moisture increase (mm), (**e**) lower-tropospheric moisture transport (streamline) and advection (mm), and (**f**) evaporation minus precipitation (mm). The boxed region [240°–270°E × 30°–45°N] in (**a**) is the main focus in the present study. This figure was created by using the Grid Analysis and Display System (GrADS) version 2.1 available at http://cola.gmu.edu/grads.

The regressed CSEOF loading vectors of PDSI and soil moisture are shown to assess the influence of warming on springtime drought (Fig. 2a). Although there are some regions with drought relief mainly in Canada and Alaska, the drought severity during March through June over central North America has generally increased due to warming (Fig. 2a). Because the PDSI takes precedent conditions into account, the overall decrease in PDSI over the central plain of North America is a robust trend. Given that the regressed field shares the same PC time series as the leading mode of 2 m air temperature (Fig. 1b), PDSI has decreased by 1–2.5 (i.e., increased aridity). The decrease in soil moisture related with boreal warming over the broad area in central North America is also found (Fig. 2b). Therefore, the central plain of North America, where the decrease in PDSI and subsurface moisture is obvious, is selected as the main target area of this study (box in Fig. 2a). The wetter condition in the Alaska region would be related to increased snow melting over high-latitude land surface.

The direction of change in humidity in warmer climate is difficult to anticipate, because the warmer air has a higher water vapor holding capability. It is often determined by two competing effects: increased evaporation due to warming and increased saturation specific humidity in accordance with the Clausius–Clapeyron equation^34^. It is therefore imperative to diagnose changes in both relative humidity and specific humidity to quantify the effect of the two competing factors separately. Detailed moisture conditions may differ from one region to another depending on the relative dominance of the two effects.

The anomaly patterns of the PDSI and soil moisture are fairly similar to that of the relative humidity (Fig. 2a–c). The reduction in relative humidity and the subsequent reduction in precipitation seems one of the primary reasons for a weak replenishment of soil moisture. This, together with the increased efficiency of evaporation due to atmospheric warming, leads to the desiccation of the soil over the central plain. The relative humidity has been reduced in the eastern Pacific and high-latitude land areas due to the warming mode, but amplitude of its reduction is particularly strong in the target region. The relative humidity has decreased by up to 5–10% considering the PC time series in Fig. 1b. Lower-tropospheric specific humidity has increased over much of North America and the neighboring oceans (Fig. 2d). There is an overall increase in specific humidity particularly at high latitudes. This increase, at least in part, may be due to the increased saturation specific humidity as a result of atmospheric warming. As noted in earlier studies, moreover, rapid sea ice melting by Arctic warming may be partly responsible for it^3,35^. However, there are some regions for decreasing specific humidity (e.g., eastern Pacific and western central North America). Both the change in water vapor amount (specific humidity) and that of temperature in the lower troposphere have contributed to an increase in the aridity over the target region (Figs 1a and 2d). The reduced water vapor content over the western central North America has resulted in decreased relative humidity at low troposphere over the target region (Fig. 2c and d).

To understand changes in the hydrological environment quantitatively, horizontal transport and vertical source/sink of moisture are examined^36,37^. Thus, the following moisture conservation equation is considered:

1 $$\frac{\partial q}{\partial t}=-\,\mathop{u}\limits^{\rightharpoonup }\cdot \nabla q+S=-\,\mathop{u}\limits^{\rightharpoonup }\cdot {\nabla }_{p}q-\omega \frac{\partial q}{\partial p}+S,$$

where *q* is specific humidity, $\mathop{u}\limits^{\rightharpoonup }$ is wind velocity, *S* is moisture source, ω is vertical (omega) velocity, *p* is pressure, and ∇_*p*_ denotes differentiation on an isobaric surface. When we multiply equation (1) by density of air (*ρ*_*a*_) and integrate with pressure coordinate, the resulting equation can be expressed as follows:

2 $$\frac{1}{{\rho }_{w}\,g}{\int }_{p}^{{p}_{0}}{\rm{\Delta }}qdp=[-\frac{1}{{\rho }_{w}\,g}{\int }_{p}^{{p}_{0}}\overrightarrow{u}\cdot {\nabla }_{p}qdp-\frac{1}{{\rho }_{w}\,g}{\int }_{p}^{{p}_{0}}\omega \frac{\partial q}{\partial p}dp+(E-P)]{\rm{\Delta }}t,$$

where *ρ*_*w*_ is density of water, *g* is gravitational acceleration, *p*_0_ is surface pressure which is assumed 1000 hPa in this study, and *E* − *P*, evaporation minus precipitation, is the net amount of moisture added to the lower troposphere. The second term on the right-hand side is negligible, so the first (horizontal transport) and the third (source) terms are investigated. From the ground perspective, signs in equation (2) are reversed (e.g., precipitation is supply of water content on the ground).

The moisture transport associated with the warming mode is examined in Fig. 2e. Two dominant anticyclonic circulation anomalies are shown over the open ocean off of the western coast and the eastern coast of North America. The anticyclonic circulation over the eastern North Pacific primarily transports moisture to the western coast of Canada, but no significant moisture transport is seen over California and the downstream of the Rocky Mountains (i.e., central plain of the United States). Although the anticyclonic flow from the Gulf of Mexico can transport the moisture to the Texas state, net moisture supply is shown to be small over the target region. Therefore, a lack of moisture transport generally is found in a large area of the central plain.

**Figure 3:**
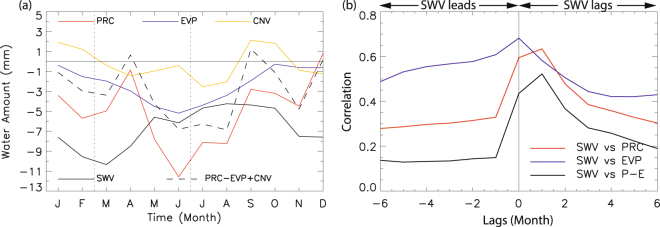
(**a**) The annual variation of various hydrological factors due to NH warming over the target domain [240°–270°E × 30°–45°N]. (**b**) Lagged correlation between soil water volume and precipitation and evaporation after removing the annual cycles.

For the moisture source and sink, change in evaporation (moisture supply to the lower troposphere) minus precipitation (moisture sink from the atmosphere) is investigated in Fig. 2f. Positive values imply that the amount of evaporation is larger and/or the amount of precipitation is smaller, so that the water content in the lower troposphere and aridity on the ground may increase, vice versa. As can be seen, net amount of moisture released from the surface via evaporation minus precipitation has increased significantly due to NH warming. It seems that the decreased soil water volume (Fig. 2b) is associated with the net increase in the amount of water released from the surface (Fig. 2f). There is increased precipitation in the west coast and the middle part of Canada, so atmospheric moisture deficit is shown in these areas. A total of approximately 40 mm of water shortage in spring (March–June) is found to have occurred in the past 38 years in the target area because of the NH warming (Fig. 3a); this amount is obtained by multiplying the March–June total atmospheric moisture deficit (PRC−EVP+CNV) in Fig. 3a with the change in the amplitude of warming in Fig. 1b (~2.8 based on the linear fitting of the PC time series). Previous researches reported that the northward expansion of the zonal band of high pressure in the subtropics would suppress rainfall under the warming situation^4–7^. Although brief heavy rainfall may have occurred more frequently in the warmer climates, it was not very helpful in terms of increasing the soil water content because of rapid surface runoff of heavy rainfalls^38^. Overall, results here suggest that the severity of drought over the central region of North America has increased due to a combination of factors including changes in precipitation or evaporation, atmospheric circulation, and land surface conditions associated with the long-term warming.

### Contributions to drought

In order to quantitatively assess the effects of meteorological variables on drought, area-averaged contributions are investigated. Figure 3a shows the yearly variations of soil moisture, moisture convergence, precipitation, and evaporation over the target region (240°–270°E and 30°–45°N) due to warming from the ground perspective. The soil moisture over the target region has decreased throughout the year (i.e., minus values for all month); judging from Fig. 1b, soil moisture has decreased by more than 20 mm in recent years compared to the early 1980s. Precipitation has been also reduced due to warming for the whole year except for December. Especially, the increased ground desiccation comes mainly from the lack of precipitation in previous months (see also Fig. 3b and supplementary material). Evaporation shows a robust reduction, which is prominent in spring and early summer (Fig. 3a). Because evaporation is closely related to sub-ground moisture condition^39^, it has also decreased simultaneously due to the increased soil dryness (Fig. 3b). It seems that different relationships of precipitation and evaporation with soil moisture make lower correlation coefficients than individual correlations (Fig. 3b). While the decreased evaporation partly compensates for the decreased precipitation, the net amount of moisture supply to the ground still has decreased during spring through early summer except for a small increase in April (dashed black line in Fig. 3a). In April, precipitation reduction is not remarkable and moisture loss due to evaporation is relatively small, and the water amount in the soil has slightly increased. Although small in magnitude, the moisture advection is also negative during March–June (Fig. 3a), indirectly contributing to the soil dryness.

While the reduction in precipitation is the largest contribution to the increased soil dryness, Fig. 2c further shows that the decrease in relative humidity is another main reason for drought over the target area. Even the evaporation has decreased due to the aridity on the ground, specific humidity on average is still slightly positive over the target area (Fig. 2d). This thermodynamic condition results from the increased saturation specific humidity related to the lower troposphere warming, leading to a decrease in relative humidity (Fig. 2c). Since saturation specific humidity increases exponentially with increasing temperature according to the Clausius–Clapeyron relation, demand for water in the atmosphere would increase rapidly, and an environment favorable for reducing soil moisture can easily be developed^40^. This situation is expected to exacerbate as warming progresses further in future^41^.

## Discussion

How drought will change in future is an issue of intense debate. Many studies have suggested that drought would become more serious with an expectation of increased dryness in future. Although we have some confidence as to the intensified future warming^42^, changes in hydrological environments are nonetheless hard to anticipate. In addition, detailed physical mechanism and drought’s contributions to environmental change still remain a challenging topic because drought is a complex phenomenon involving various climatological elements such as warming, soil moisture, precipitation, evaporation, and moisture transport. This study shows a diagnosis of how the climatological factors vary due to NH warming via CSEOF analysis in order to assess quantitative change in drought and hydrological environments. On the basis of this understanding, it is suggested that drought in spring through early summer over the central plain of North America, a main crop producing region, will become more severe in future if NH warming continues to intensify^41,42^. This is an interesting result, since significant warming has mainly occurred in the high-latitude region during the cold season. Although drought may be influenced by different types of variability on various time scales, warming-related change will be a dominant factor in future. Considering the physical mechanism mentioned above, drought over the central plane of North America will pose a major problem for food security in the world^20–22^.

Regarding the environmental changes associated with warming, the decreased soil moisture can amplify the impact of heat waves^43^. Heat waves develop more easily on dry lands. Intensified heat waves will lead to severer or longer drought in future. In addition, it is also possible that mountain snow melts earlier due to warming, and water supply to a target region may significantly vary^19^. Expansion of the tropical latitude band due to warming is also an important factor in that it can change both the distribution and amount of precipitation. Many, if not all, of these factors may affect soil moisture and ultimately drought in a future warmer climate.

There are still lingering questions as to how the drought characteristics such as the onset and termination conditions and duration will change with warming. Since the effect of drought should be considered in a cumulative manner, it is rather difficult to address the drought characteristics in the present study. Under continued global warming, it would be more important to understand the detailed mechanism of drought development and assess drought risk from a climatological point of view. Accordingly, future study should elucidate the change in detailed evolution of drought and duration. This study may be regarded as comprehensive view on boreal temperature warming and the mechanism of drought in North America.

Finally, uncertainty is inherent in the present analysis, since reanalysis datasets are not perfect^44,45^. In order to address this issue, we repeated similar calculations based on two other reanalysis products: Modern-Era Retrospective Analysis for Research and Applications (MERRA)^46^ and National Center for Environmental Prediction/National Center for Atmospheric Research (NCEP/NCAR)^47^. The two reanalysis products show qualitatively similar changes of hydrological variables to those in Fig. 2. On the other hand, the magnitude of change differs somewhat from one reanalysis dataset to another. Therefore, we think that the water budget change described in Fig. 2 is valid but are less confident on the magnitude of the hydrological changes over the target area.

## Methods

### Reanalysis datasets

The variable used in this study are 2 m temperature, 3-dimensional relative humidity, specific humidity, and wind, precipitation and evaporation at the surface, and soil moisture for the first three layers (root-zone, top-1 m) from the monthly ERA-interim reanalysis product at a 1.5-degree resolution for the period of 1979–2016^48^. To assess the drought severity, self-calibrated PDSI monthly dataset during the period of 1979–2014 is used^32,33^. This dataset was obtained from the Research Data Archive at the National Center for Atmospheric Research. PDSI is a relative drought index describing dryness compared to a reference period by measuring the water balance between moisture demand (potential evaporation) and supply (precipitation) on the ground. To obtain the potential evaporation, the Penman-Monteith equation, accounting for the effects of available energy, humidity, and wind speed, is used^49^. It is known that the Penman-Monteith equation minimizes the PDSI calculation error in energy-limited regions^49^. In addition, self-calibrating PDSI can be produced by incorporating local conditions into the original PDSI. It is usually used for assessing long-term drought characteristics and agricultural impacts. The self-calibrated PDSI index automatically adjusts its behavior at every position by substituting the dynamically calculated value for the empirical constant in the index calculation^50^.

### Cyclostationary Empirical Orthogonal Function analysis

CSEOF analysis is applied to extract physically evolving climate variability^29,30^. Let *T*(*r*, *t*) be a climate variable, then principal modes of space-time evolution patterns are extracted as:

3 $$T(r,\,t)=\sum _{n}L{V}_{n}(r,\,t)P{C}_{n}(t),\,\,L{V}_{n}(r,\,t)=L{V}_{n}(r,\,t+d),$$

where *n* is the CSEOF mode number, *r* and *t* denote location and time, *LV*_*n*_ (*r*, *t*) is the *n*th CSEOF loading vector, and *PC*_*n*_ (*t*) is corresponding PC time series. An advantage of CSEOF analysis is that it takes into account of temporally varying spatial patterns. Because of the cyclostationarity assumption, CSEOF loading vectors are periodic with the periodicity *d*, which is referred to as the nested period. The nested period is set to 12 months in the present study because the statistical properties (mean and covariance) of the climate variables including the 2 m air temperature exhibit one-year periodicity.

After CSEOF analysis is conducted on all variables, regression analysis in CSEOF space is carried out to make CSEOF loading vectors from different variables physically and dynamically consistent^51^. The specific goal of the regression analysis in CSEOF space is to make loading vectors of predictor variables (PDSI, soil water volume, relative humidity, specific humidity, moisture convergence, and moisture source/sink) share the same PC time series with the target variable (2 m air temperature in this study). Then, the loading vectors derived from different variables are considered to be physically consistent with each other including the target variable. As the first step, CSEOF PC time series of each predictor variable, *PCP*_*m*_ (*t*), is regressed onto target PC time series, *PC*_*n*_ (*t*), as follows:

4 $$P{C}_{n}(t)=\sum _{m=1}^{M}{a}_{m}^{(n)}PC{P}_{m}(t)+{\varepsilon }^{(n)}(t),$$

where *M*, ${a}_{m}^{(n)}$, and *ε*^(*n*)^ are the number of predictor PC time series, regression coefficients, and regression error, respectively. The regression coefficients are determined such that error variance is minimized. Then, the regressed CSEOF loading vectors for the predictor variables, *LVPR*_*n*_ (*r*, *t*), are determined by:

5 $$LVP{R}_{n}(r,\,t)=\sum _{m=1}^{M}{a}_{m}^{(n)}LV{P}_{m}(r,\,t),$$

where *LVP*_*m*_ (*r*, *t*) are the original CSEOF loading vectors for the predictor variable. As a result of regression analysis in CSEOF space, the entire variables used in this study can be written as

6 $$Data(r,\,t)=\sum _{n}\{{T}_{n}(r,\,t),\,{R}_{n}(r,\,t),\,{U}_{n}(r,\,t),\,{V}_{n}(r,\,t),\ldots \}P{C}_{n}(t),$$

where {*T*_*n*_ (*r*, *t*), *R*_*n*_ (*r*, *t*), *U*_*n*_ (*r*, *t*), *V*_*n*_ (*r*, *t*), …} refer to the (regressed) CSEOF loading vectors which show how the space-time evolution is manifested in different variables in a physically consistent manner for each mode *n*. All the loading vectors in (6) are governed by the PC time series of the target variable (2 m air temperature). The loading vector *T*_*n*_ (*r*, *t*) of the target variable (2 m air temperature) is depicted in Fig. 1 and the regressed loading vectors {*R*_*n*_ (*r*, *t*), *U*_*n*_ (*r*, *t*), *V*_*n*_ (*r*, *t*), …} of the predictor variables are depicted in Fig. 2 (as March–June averages) and Fig. 3 (as averages over the target area) for *n* = 1 (referred to as the warming mode in the present study). A proof of (6) being derived from (4) and (5) is mathematically involved and is considered beyond the scope of the present study^52^.

## Electronic supplementary material

Supplementary Material
