# Supplementary material for: Physical mechanism of spring and early summer drought over North America associated with the boreal warming
Source: Sci Rep. 2018 May 14;8:7533. doi: 10.1038/s41598-018-25932-5 (PMC5951915; doi:10.1038/s41598-018-25932-5)
Supplement: Supplementary file 1 — Supplementary Material [file 41598_2018_25932_MOESM1_ESM.pdf]

Physical mechanism of spring and early summer drought over North America associated with the boreal warming

Woosuk Choi and Kwang-Yul Kim*

School of Earth and Environmental Sciences, Seoul National University, Seoul, Korea

Supplementary materials

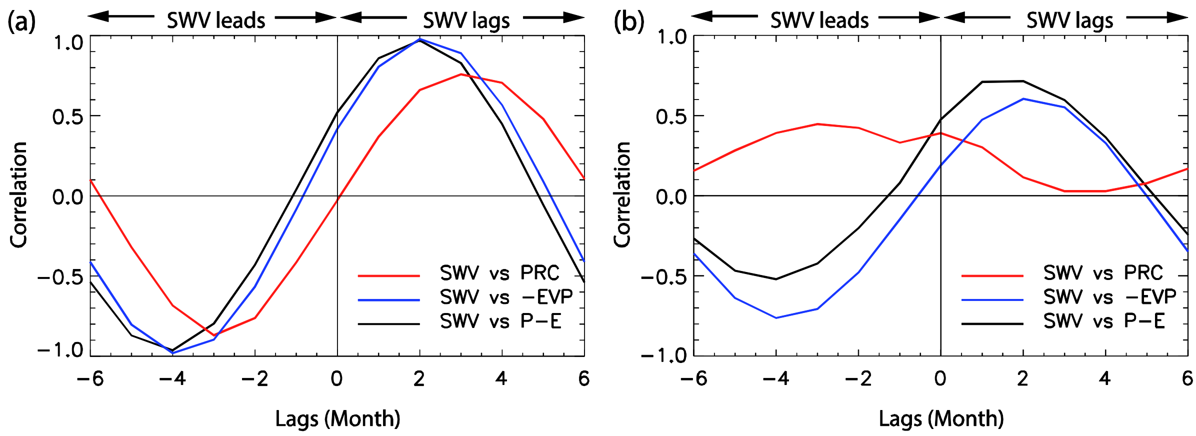

Figure S1. (a) Lagged correlation between the annual cycles of soil water volume and those of precipitation and evaporation. (b) Lagged correlation between soil water volume and precipitation and evaporation. The time series are the averages over the domain [240°–270°E × 30°–45°N].

Figure S1a shows that soil water volume lags evaporation by two months and precipitation by three months within the annual cycle. Overall, moisture source term (precipitation minus evaporation) leads soil moisture by about two months. Thus, in the context of the annual cycle, the moisture supply two months earlier is a dominant factor in the water content in the soil. However, correlation coefficients decrease in the raw time series (Fig. S1b), particularly correlation between soil water volume and precipitation. This indicates that the annual variation of precipitation differs significantly from the variation of soil moisture, while the variation of evaporation looks similar to that of soil moisture.

In the absence of the annual cycles, soil water volume is positively correlated with and slightly leads evaporation (Fig. 3). Thus, the decreased soil water volume leads to the decreased evaporation. Precipitation is positively correlated with soil water volume at 1-month lag; thus, a decrease in precipitation results in a reduction of soil water volume, and

vice versa. The reduced net water supply to the soil seems to be responsible for the reduced soil moisture. That is, a reduction in the net supply of water to the soil via decreased precipitation is the primary reason for the reduction of soil moisture. On the other hand, moisture content in the lower troposphere increases, which is reflected in the increased specific humidity. Thus, the decreased precipitation is primarily due to the increased specific humidity associated with the tropospheric warming; this is also reflected in the decreased relative humidity over the target region.

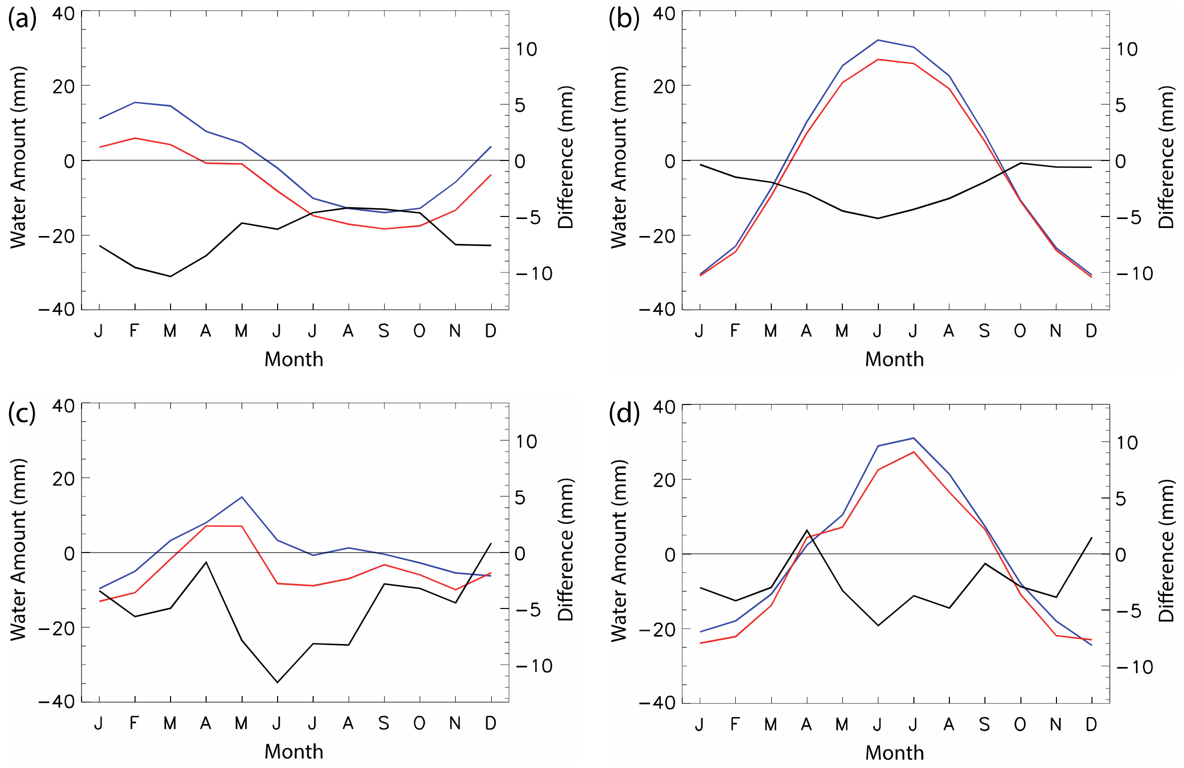

Figure S2. Annual cycles of (a) soil water volume, (b) evaporation, (c) precipitation, and (d) precipitation minus evaporation. Blue lines are the annual cycle of each variable and red lines are the sum of annual cycle and warming mode variation. Black curves show the difference between blue and red lines, which means variation of warming mode, the same as lines in Fig. 3.

As can be seen in Fig. S2, soil water volume has decreased more significantly in spring whereas the precipitation and evaporation has decreased more significantly in summer. Evaporation decreases throughout the year depending on the warming mode (Fig. S2b). Precipitation has been reduced by warming mode except for December, and maximum reduction is shown by June (Fig. S2c). The net amount of moisture supply to the soil is negative except in April and December (Fig. S2d). This implies that the soil loses water due to NH warming, and this net loss is primarily due to the decreased precipitation.
